# The Surgical Treatment of Hypertension—I
*See footnote, page 103.


**Published:** 1950-10

**Authors:** G. L. Alexander

**Affiliations:** Neurological Surgeon, Bristol United Hospitals


### THE SURGICAL TREATMENT OF HYPERTENSION—I *

BY

G. L. ALEXANDER, F.R.C.S.
*Neurological Surgeon, Bristol United Hospitals.*

---

It seemed pertinent to this discussion to inquire into the after-histories of patients operated on in Edinburgh after an arbitrarily-determined period of five or more years. The series of eighteen patients from Professor Dott's material which fall into that category have been re-examined by me in the past few months or have reported personally by letter or through their medical attendant. The cases in this series form part of the basis of a recent paper by Dr. A. R. Gilchrist and his co-workers. All of the patients were assessed before and after operation by Dr. Gilchrist and his staff. Thus there was some measure of uniformity in selection and assessment, without bias in favour of surgery.

Of the eighteen patients half were men. Four were over fifty years of age. All came for advice because of disabling symptoms, and headache was the predominant complaint. What I might term the " settled " diastolic pressure—after some weeks of rest —was 130 mm. or more in eleven of the sixteen cases of benign essential hypertension.

There was one *known death* in the series; she died with papilloedema and encephalopathy four years after sympathectomy. A second patient, now untraceable, has *probably died;* two years after operation she had encephalopathy, and retinae were grade III.[1] Two others, of no fixed address, cannot now be traced. They were followed in good health for one and five years respectively.

One half of the eighteen patients have been seen by me personally in recent months. Not one of those fourteen of which we

* See footnote, page 103.

have up-to-date information is an invalid by reason of the disease and the majority are leading active lives.

### Malignant Hypertension

Two cases fall into this category. The first was a girl of nineteen years, with S–T changes in electrocardiogram. She had a purely subdiaphragmatic operation (Adson type) eleven years ago. Her blood pressure, which had remained around 204/124 mm. during six months of observation dropped to 130/90. Subsequently she married, and there was a still-birth with eclampsia. Her kidneys were considered to have fully recovered two years later. She had an uneventful pregnancy in 1945, this time well-supervised, six years after her operation. During this pregnancy there was a trace of albumin in the urine; her blood pressure never exceeded 150/90 mm.

In 1946 her blood pressure had risen to 200/140 mm. She was having headaches again but her E.C.G. was normal; (Dr. Davies has seen the prints and corroborates this). The retinal arteries were now quite tortuous but there was no papilloedema.

Re-operation was undertaken on one side, this time by Smithwick procedure, the chain from T7–L3 being removed together with the splanchnic nerves above the diaphragm. We observed at operation that there was a limited attempt at regeneration from the lower end of the greater splanchnic nerve, divided at the crus in the first operation. A nerve bundle about 1.5 mm. in diameter, of the appearance and consistence of sympathetic nerve, passed round the silver clip which we had earlier applied to the great splanchnic nerve before section, and this nerve bundle proceeded caudally for about 2 cm. to become lost in connective tissue in relation to the aorta. It is regrettable that this tissue was lost and microscopic examination was thus denied.

Several observers agreed during follow-up in the next year that the retinal arteries became less tortuous. The E.C.G. has remained normal and a recent blood-pressure reading was around 150/90 mm. This patient goes to dances, manages a household with three young children (two step-children) and for periods has no domestic help. She is having mild headache and is at times irritable. Further extensive sympathectomy will probably be necessary on the other side at some future date.

The second malignant hypertensive patient, a man of forty-five years, attracted our attention because he first demonstrated to us the rapidity with which papilloedema and albuminuria could subside in the two weeks or so between the two operations. He was operated on six years ago. Last year this man was re-admitted to hospital with *acute recurrence* of encephalopathy and papilloedema; he had once again defective renal excretion and gross T-wave changes. He returned to his very strenuous business life (7.30 a.m.—6.30 p.m.) after three months of rice diet. When seen recently by me his pressure was 230/140 mm. (as when first seen), the retinae are Grade II and the urine shows slight albumen and some granular casts. This operation I should now consider to have been incomplete. I found the anatomy of the thoracic sympathetic difficult to identify on both sides and I have little doubt now that a substantial para-aortic structure remained untouched.

### Essential Hypertension

The rest of the patients fall into this category. One deserves special mention as she was probably about to slip into the malignant phase. She was an Irish lady of thirty-nine years. There was an atrocious family history. She was the sole survivor of a family of five, each of whom had died between the ages of forty and forty-two with hypertension and cerebral haemorrhage, angina or uraemia. She had a long history of disabling symptoms but only a Grade I retina; her pressure was 210/120 mm. We operated on her eight years ago. We have no recent reports on the state of her retina or E.C.G. She leads an active social life, and rests (perhaps) an hour a day, but offsets that by smoking excessively. She is now forty-seven years of age. Her diastolic pressure has been climbing very slowly to 120 mm.

Thus the three cases of the series with the worst pre-operative prognosis are still alive and active eleven, six and eight years respectively after sympathectomy.

What of the eleven others with essential hypertension of whom we have up-to-date knowledge? I am afraid I cannot answer the question whether their operations were really necessary—which is what we really want to know. With one exception the patients are all leading active lives. Two reached retirement at the age of sixty years: but one does part-time work as an insurance agent, with a lot of stair-climbing involved, and both play several rounds of golf weekly. One other spent the war years instructing Commandos in unarmed combat, and now chases salmon poachers. Another was from 1943–49 the general manager of a large marine repair-yard on the Clyde: yet another has enlarged his small garage business to an engineering concern employing 600 hands. One naval officer of high rank was retired because of his disability; he has had anginal symptoms in the past two years but takes nitroglycerin and refuses to rest. And there are three housewives; in these days one need say no more. The one exception referred to above concerns a lady who showed slight psychotic tendencies at the time of her operations seven years ago. She has retired comfortably into a mental hospital and spends—in her case—a placid existence, with her diastolic pressure consistently 40 mm. lower than before operation. The placid existence is significant.

What has happened to the blood-pressures of those patients in the group of benign essential hypertension? I find that the diastolic pressure is now 20 mm. or more lower than before operation in seven cases out of the eleven. In two instances the diastolic pressure is the same as before operation, and one of those is the Irish lady with the family history of grave cardio-vascular disease. In three cases there has been a slow fall in diastolic pressure over the past three years and in the four others the pressure remained lowered since immediately after operation.

Keith, Woolf and Gilchrist[2] recently surveyed 150 cases of essential hypertension, of which fifty-five had been subjected to operation. They found that in thirty-one operated cases suitable for analysis the mean *lowering* of diastolic pressure one year after operation was 8 mm. A comparison with carefully-matched cases treated medically showed a mean *rise* of 8 mm. in the diastolic pressure after one year of observation. They had altogether forty-two medical and forty-seven surgical cases suitable for statistically-acceptable comparison. These were divided into eleven sub-groups comparable in retinal grading, cardiac and renal efficiencies, severity of symptoms, and diastolic pressure. Their statistician collaborator imposed rigid controls on their workings. They found that although the *mortality-rates* in the medical and surgical sub-groups were similar the *work-capacity*

of the surgically-treated patients after one year was decidedly better than in the medical group. E.C.G. improvement was seen in about one-fifth of the operated group with pre-operative abnormalities. None of the medical group responded in this way.

Gilchrist recommends that estimations of reduction in size of the heart should be deferred until at least six months after operation, so as to eliminate the effect due to postural hypotension. He found that there was a 10 per cent. reduction in frontal cardiac area in normal subjects with nitroglycerin. He has been using the method of Ungerleider and Gubner[3] in assessing cardiac area on films

With reference to postural hypotension it is interesting to record that the marine engineer patient on a visit to the United States in 1946, three years after his operations, experienced a " G-effect " in the express elevators as they came to a stop during descent. Gilchrist and his colleagues consider that the amytal test is not a useful index of probable relief of symptoms but is a guide in forecasting postural hypotension after operation.

Some years ago we had come to agreement that the indications for sympathectomy were severe symptoms interfering with daily life, such as headache, impaired intellectual capacity and stamina, irritability, and the finding of retinal spasm, in patients not in general exceeding fifty-five years of age. Contra-indications are nitrogen retention, oedema, and congestive heart failure. The blood urea-nitrogen figure is not reliable as an index of renal efficiency; the van Slyke urea clearance test is preferred. In regard to *operative mortality* I have had personal contact to date with forty-four cases. There was one (avoidable) death from septicaemia due to an operative infection, occurring in the days before penicillin.

I am much indebted to Professor Norman Dott, C.B.E., for encouraging me to quote from his material. Dr. A. R. Gilchrist and his associate Dr. M. Keith have given much help and information for which I am grateful. Dr. Gibbs was also of assistance in obtaining copies of E.C.G. for study, and in other ways.

REFERENCES.

1. Wagener, H. P., and Keith, N. M. (1939).—*Medicine*, **18**, 317.
2. Keith, M. A., Woolf, B., and Gilchrist, A. R. (1949).—*Brit. Heart J.*, XI, No. 3, 287.
3. Ungerleider, H. E., and Gubner, R. (1942).—*Amer. Heart J.*, **24**, 494.

